# A prospective, multicentre study to investigate the efficacy, safety and tolerability of octreotide LAR® (long-acting repeatable octreotide) in the primary therapy of patients with acromegaly

**DOI:** 10.1111/j.1365-2265.2007.02825.x

**Published:** 2007-06-01

**Authors:** Moises Mercado, Fatima Borges, Hakim Bouterfa, Tien-Chun Chang, Alberto Chervin, Andrew J Farrall, Attila Patocs, Stephan Petersenn, Jan Podoba, Mitra Safari, Joanna Wardlaw

**Affiliations:** * Hospital de Especialidades, Centro Medico Nacional Siglo XXI, IMSS Mexico City, Mexico; † C2 Hospital de Santo António, Porto Portugal; ‡ Novartis Pharma AG, Basel Switzerland; § National Taiwan University Hospital Taipei, Taiwan; ¶ Hospital Santa Lucia, Buenos Aires Argentina; ** University of Edinburgh Edinburgh, UK; †† Semmelweis University, Budapest Hungary; ‡‡ Universitaetsklinikum, Essen Germany; §§ Interna Klinika FNsP Bratislava, Slovakia

## Abstract

**Objective:**

To evaluate the efficacy, safety and tolerability of octreotide LAR® (long-acting repeatable octreotide) in the primary therapy of acromegaly.

**Design and patients:**

Ninety-eight previously untreated acromegalics were recruited into this prospective multicentre study. A total of 68 patients successfully completed 48 weeks of the study period, received 12 doses of octreotide LAR 10–30 mg every 4 weeks, and constituted the population used for this analysis.

**Measurements and results:**

A clinically relevant reduction (i.e. to ≤ 5 µg/l) in mean GH (mGH) was recorded in 72% of patients after 24 weeks of treatment, and 42% reached a ‘safe’ GH value (≤ 2·5 µg/l). At week 48, 16 more patients were considered partial GH responders (GH > 2·5 µg/l and ≤ 5 µg/l) and 44% had reached a GH level ≤ 2·5 µg/l. IGF-1 levels normalized in 38% and 34% of patients after 24 and 48 weeks of treatment, respectively. At study completion, 10 patients (14·7%) who had not normalized their IGF-1 levels had achieved at least a 50% decrement in this marker. In eight microadenoma patients, tumour volume decreased from a mean baseline level of 298 ± 145 mm^3^ to 139 ± 94 mm^3^ after 24 weeks and to 99 ± 70 mm^3^ after 48 weeks of therapy. In 60 patients with macroadenoma, the corresponding values were 3885 ± 5077 mm^3^ at baseline and 2723 ± 3435 and 2406 ± 3207 mm^3^ after 24 and 48 weeks, respectively. At weeks 24 and 48, a significant (> 20%) tumour volume reduction was reported in 63% and 75% of patients, respectively. A reduction in the severity of symptoms of acromegaly was observed early in treatment and was maintained throughout the study period.

**Conclusion:**

Octreotide LAR represents a viable alternative to surgery for primary treatment of acromegaly leading to a progressive regression of tumour volume, a sustained control of biochemical abnormalities and an adequate relief of symptoms of the disease.

## Introduction

Acromegaly results from an excess secretion of GH from a pituitary adenoma leading to an increase in IGF-1 concentrations.^1^,^2^ The symptoms are due to the effects of both GH and IGF-1 on cartilage, soft tissues and organs, such as the heart, and the compression of the surrounding tissues by the tumour itself, which results in headache, visual field defects and hypopituitarism.^1^,^2^ Patients with active acromegaly have an elevated morbidity and mortality that correlate with the elevated plasma levels of GH and IGF-1.^3^,^4^

The principal objectives in the treatment of acromegaly are: (1) to halt or reverse tumour growth, thereby controlling symptoms of compression, (2) to improve symptoms and comorbidities resulting from the excessive plasma concentrations of GH and IGF-1 and (3) to eliminate the increased mortality rate associated with the disease by effectively reducing GH and by normalizing IGF-1 concentrations.^5^–^7^

Although surgical remission rates achieved in patients with microadenomas are high, provided an experienced pituitary neurosurgeon performs the procedure (depending on the definition of cure, 80–90%),^8^,^9^ over 70% of acromegalic patients harbour macroadenomas and have surgical cure rates of less than 50%, even in the most experienced hands.^10^,^11^ However, the frequent presence of tumour in inaccessible sites such as the cavernous sinuses can make surgical attempts futile. Therefore, for a significant proportion of patients, surgery does not represent a satisfactory form of therapy.

Primary pharmacological therapy of patients with acromegaly with depot somatostatin analogues can be effective, both in terms of GH and IGF-1 control and in reducing tumour volume.^12^–^16^ In general, these series report biochemical success rates (achievement of a GH value < 2·5 µg/l and a normal IGF-1 level) that range between 40% and 70%. The present multicentre, international study was undertaken to evaluate the efficacy and safety of octreotide LAR® (long-acting repeatable octreotide) in a rigorously controlled, prospective setting, with particular emphasis on accurate tumour volume measurements and their correlation with clinical and biochemical outcome.

## Patients and methods

This prospective, open-label international multicentre study was carried out at selected neuroendocrinological clinics to investigate the feasibility of using octreotide LAR administered by intramuscular (i.m.) injection at intervals of 4 weeks, as the primary medical therapy of previously untreated acromegalic patients. A total of 31 centres in 15 countries participated, recruiting patients into the study between December 2002 and August 2003.

### Patients

Male or female patients aged 18–80 years with previously untreated acromegaly who provided their written informed consent were eligible to participate in the study. Biochemical diagnosis of acromegaly required both a lack of GH suppression to < 1 µg/l after a 75-g oral glucose load and an elevated IGF-1 to above the 97th percentile of the normal range adjusted for age and gender.

Excluded from the investigation were patients who had received any prior therapy for acromegaly, those with significant deterioration of visual fields or other neurological signs related to the tumour mass or significant medical conditions that may have interfered with the study. Also excluded were patients in whom magnetic resonance imaging (MRI) revealed no evidence of a pituitary adenoma, those being treated with any drug for the inhibition of prolactin secretion or patients with a history of symptomatic cholelithiasis. The experimental protocol was approved by the independent ethical review committees of each centre participating in the study.

### Study design

At the screening visit blood was collected for GH measurement at 0 (after 1 h rest), 30, 60, 90 and 120 min and, subsequently, a subcutaneous (s.c.) 100 µg octreotide test dose was administered to ensure that the drug was tolerated and additional blood samples were collected 30, 60, 90 and 120 min later. Selection of patients was not based on the responses to the s.c. octreotide test. Patients who fulfilled all the criteria for study participation then commenced therapy with octreotide LAR 20 mg i.m. At week 12, after three doses of octreotide LAR had been administered, mean GH (mGH) and IGF-1 were measured prior to the fourth injection. At week 16, dose titration was permitted and in patients in whom mGH levels were < 1 µg/l and IGF-1 was within the adjusted normal range the dose was decreased to 10 mg, those in whom the mGH was ≤ 2·5 µg/l and the IGF-1 was within the adjusted normal range continued to receive 20 mg and in those in whom GH was > 2·5 µg/l or the IGF-1 above the adjusted normal range the dose was increased to 30 mg. If necessary, further dose adjustment was permitted after 24 weeks of therapy (at the week 28 visit).

### Assessment of biochemical response

Serum GH and IGF-1 levels were measured at a central laboratory (Nichols Institute Diagnostics, San Clemente, CA, USA) by immunoradiometric assay (IRMA). For GH, the lower limit of detection was 0·5 µg/l and the within-run coefficients of variation (CVs) ranged from 4·2% to 4·6% and between-run CVs ranged between 5·0% and 5·4%. For IGF-1 measurements, serum samples were pretreated with acid–ethanol to allow for binding protein separation prior to the assay, which had a detection limit of 30 µg/l and intra- and interassay CVs of 6·2% and 6·1%, respectively.

Biochemical outcome categories were defined as follows:

- ‘Total success’ was achieved when the mGH was ≤ 2·5 µg/l and age-adjusted IGF-1 was normal.
- ‘Partial success’ was defined as: (1) an mGH > 2·5 µg/l and ≤ 5·0 µg/l and a decrease in IGF-I of ≥ 50% compared to baseline, or IGF-1 within the age-adjusted laboratory normal range, or (2) an mGH ≤ 2·5 µg/l and a decrease in IGF-I of ≥ 50% compared to the baseline level, but IGF-I levels still above the normal range.
- ‘Treatment failure’ was defined as any other response.

### Symptoms of acromegaly

Patients were asked to score each of their symptoms of acromegaly (0 = absent, 1 = mild, 2 = moderate, 3 = severe but not incapacitating or 4 = severe and incapacitating) at baseline and at 16, 24 and 48 weeks of therapy. The symptoms scored were headache, fatigue, perspiration, paresthesia, osteoarthralgia and carpal tunnel syndrome.

### MRI studies

MR images were acquired at each participating site, based on the optimum imaging protocol used in each centre, but included as a minimum, T1-weighted sagittal and coronal sequences before and after administration of gadolinium (in a standard diagnostic imaging dose). The minimum scanner field strength was 1·0 Tesla. All image data were sent electronically (when possible) or printed onto film to a central image reading laboratory for blinded analysis.

The pituitary tumour measurements from the MRI scans at baseline, week 24 and week 48 were made in a central laboratory by three neuroradiologists blinded to all clinical and endocrine data. Pituitary lesion images received electronically from participating centres in digital (DICOM) format were loaded directly into the image analysis and handling software (Digital Jacket, Version 3·6; DesAcc Inc., Chicago, IL, USA). Images received as film were redigitized with a film scanner (Multirad 450, Howtek, OH, USA) and converted to DICOM format using Digital Jacket. The coronal images were used for tumour volume measurements. Images were magnified by a factor of 2; tumour circumferences were outlined using the polygonal outline tool on each slice on which the tumour was visible, the area within the circumference on each slice was calculated by the Digital Jacket program, and the thickness of each slice (slice thickness + any gap spacing) was also recorded. Tumour volume was calculated as the sum of each tumour slice area multiplied by the effective section thickness.^17^ Maximum tumour diameter was determined from the largest diameter in any plane. Tumours were classified as micro- or macroadenomas based on the largest tumour diameter (≤ 10 mm or > 10 mm, respectively).

Estimates of the accuracy and reproducibility between patient visits due to, for example, variations in patient positioning, contrast bolus timing and volume averaging artefacts cumulatively contributed to a variation of at least 20%. Thus, a change in tumour volume was only considered significant if it exceeded 20%.

### Gallbladder ultrasound evaluation

Ultrasound evaluations of the gallbladder and biliary tree were performed at baseline and after 24 and 48 weeks of therapy with the study drug. The results of these investigations were assessed and interpreted locally.

### Statistical analysis

The rate of treatment success and the corresponding 95% confidence interval was calculated using the normal approximation to the binomial distribution, applying a continuity correction: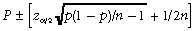 where *P* represents the observed proportion of treatment success, *z*_α/2_ is the 100(1 − α/2) percentile of the normal distribution, *n* represents the number of patients and α = 0·05. For the calculation of the confidence intervals in the microadenoma patients, the method of Clopper and Pearson^18^ was used, in consideration of the small number of subjects in this group. Nonparametric Wilcoxon two-sample tests (based upon rank sums) were applied to examine group differences for continuous variables. To compare fractions between categorical variables, χ^2^-tests or Fisher's exact test (in the case of small samples) were used. A *P*-level of 5% or less was considered as statistically significant.

## Results

Out of a total of 110 patients screened for acromegaly, 98 fulfilled the protocol inclusion criteria and were started on octreotide LAR after their informed consent had been obtained. Thirty patients were excluded from the efficacy analysis of this report, 15 because of major protocol violations and 15 because of premature discontinuation. Of the 15 patients who were prematurely discontinued from the study, six discontinued as permitted by the protocol due to uncontrolled acromegaly (GH > 2·5 µg/l and/or IGF-1 above the upper limit of normal (ULN) for age), one withdrew consent and eight patients withdrew because of adverse events (AEs). Of the latter, two cases (one pregnancy with spontaneous abortion and one case of pancreatic carcinoma) were considered to be unrelated to therapy whereas the remaining six (abdominal disturbances with or without cholelithiasis) were suspected of being drug induced. Thus, 68 patients (eight with microadenomas and 60 with macroadenomas) completed the full course of the investigation, receiving 12 injections of the study drug, and constitute the per protocol population that is the subject of this report. Their baseline clinical, biochemical and tumour volume by MRI characteristics are presented in Table 1. Age, gender proportion, disease duration, tumour size and biochemical severity were no different between the 30 subjects who had to abandon the study and the 68 who completed it. Diabetes mellitus was present in 18% of the patients at study entry. Thirty-four patients (35%, all macroadenomas) showed mild hyperprolactinaemia at the beginning of the study; of these, seven (7%) normalized their prolactin while on treatment with octreotide LAR.

**Table 1:** Baseline characteristics

	Total *N* = 68	Microadenomas *N* = 8	Macroadenomas *N* = 60	P*
Age (years)				
Mean ± SD	49·7 ± 13·2	60 ± 9·3	48·3 ± 13·1	
Median	50	57·5	47·5	0·02
Range	24–77	49–77	24–71	
Male/female	28/40	4/4	24/36	0·7
mGH (µg/l)				
Mean ± SD	33·6 ± 62·6	5·5 ± 2·6	37·4 ± 65·8	
Median	9·5	5·3	13·1	0·008
Range	2–391	2·3–9·1	2–391	
IGF-1(µg/l)				
Mean ± SD	676 ± 275	570 ± 124	690 ± 287	
Median	616	565	617	0·19
Range	277–1974	391–720	277–1974	
Tumour volume (mm^3^)				
Mean ± SD	3463 ± 4905	298 ± 145	3885 ± 5077	
Median	1210	308	1608	< 0·0001
Range	59–22 702	86–528	59–22 702	

* Wilcoxon test for continuous variables, Fisher's exact test for gender.

### Drug doses

At the end of the study, among the patients with microadenomas, one was receiving 10 mg, three were on 20 mg and four on 30 mg. In the group with macroadenomas, four patients were treated with 10 mg, 12 with 20 mg and 44 with 30 mg.

### Biochemical responses

The effects of octreotide LAR on the mean and median serum levels of mGH and IGF-1 after 12, 24 and 48 weeks of treatment are summarized in Table 2. The majority of subjects showed a beneficial effect, with substantial decreases in the serum concentrations of both GH and IGF-1 noted as early as 12 weeks after the beginning of therapy. In patients with microadenomas, the average reduction in mGH after 24 and 48 weeks of octreotide therapy was 71·2% and 69%, respectively. In patients with macroadenomas, the average percentage of decrement in mGH was 70·8% and 76% at weeks 24 and 48 of the study, respectively. The average decrease in IGF-1 levels was of a smaller magnitude: 66% and 61·7% for microadenomas at weeks 24 and 48, respectively, and 42% and 37% for macroadenomas at the same time points. The effects of treatment on serum concentrations of GH and IGF-1 in individual patients are illustrated inFigs 1 and 2, respectively.

**Fig. 1:**
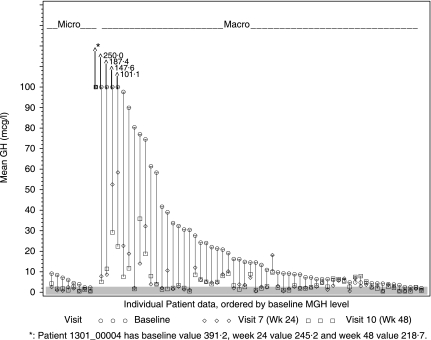
Mean GH values in each individual patient at baseline, and after 24 and 48 weeks of treatment with octreotide LAR. Shaded area represents a GH level < 2·5 µg/l.

**Fig. 2:**
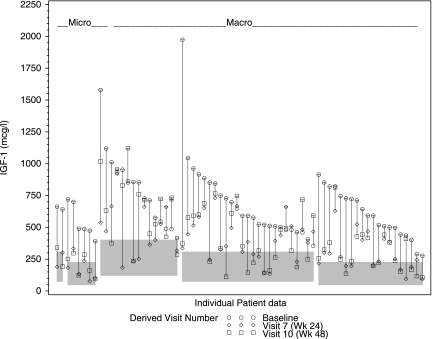
Individual IGF-1 levels at baseline and after 24 and 48 weeks of treatment with octreotide LAR. Shaded areas represent the normal, age-adjusted range.

**Table 2:** Mean GH (mGH) and IGF-1 at the different follow-up visits

		Visit				Difference
			
		Baseline	Week 12	Week 24	Week 48	Baseline – Week 48
*Microadenoma*						
	*N*	8	8	8	8	8
mGH (µg/l)	Mean	5·5	1·6	1·5	1·7	−3·8
	Median	5·3	1·6	1·4	1·5	−4·1
	Min, Max	2·3, 9·1	0·5, 3·1	0·5, 2·6	0·5, 4·2	−6·7, −1·1
	SD	2·6	1·0	0·8	1·2	2·0
IGF-1 (µg/l)	Mean	570·6	210·5	194·1	218·4	−352
	Median	565·0	222·5	186·0	222·0	−344
	Min, Max	391, 720	86, 354	77, 334	95, 340	−468, −203
	SD	123·9	97·0	92·0	88·3	87·4
*Macroadenoma*						
	*N*	60	60	60	60	60
mGH (µg/l)	Mean	37·4	14·3	10·9	9·0	−28·3
	Median	13·9	3·0	3·2	3·3	−9·1
	Min, Max	2, 391·2	0·5, 401·4	0·5, 245·2	0·5, 218·7	−245, 3·2
	SD	65·8	52·5	32·6	28·3	47·6
IGF-1 (µg/l)	Mean	690·3	396·2	399·2	435·6	−255
	Median	617·5	345·5	383·0	381·5	−236
	Min, Max	277, 1974	93, 818	92, 922	107, 1122	−1602, 258
	SD	287·7	199·3	192·4	248·3	296·0

A GH level of 2·5 µg/l or below was reached by 42·6% of patients (87·5% with microadenomas, 36·7% with macroadenomas) after 24 weeks of therapy, whereas the IGF-1 level had normalized in 38·2% (75% with microadenomas and 33·3% with macroadenomas) of treated subjects at this time point (Table 3). The proportion of patients with a complete GH and IGF-1 response did not increase over time (44·1% and 33·8%, respectively, at 48 weeks) (Table 3). At the end of the study 25% of the patients had simultaneously met both GH and IGF-1 criteria for treatment success (Table 4).

**Table 3:** GH and IGF-1 response rates after 24 and 48 weeks of treatment

Week/Outcome		Total *N* = 68% (*n*) [95% CI]	Microadenomas *N* = 8% (*n*) [95% CI]*	Macroadenomas *N* = 60% (*n*) [95% CI]
Week 24				
Any GH response		72·1 (49) [60·6, 83·5]	100·0 (8) [63·1, 100·0]	68·3 (41) [55·6, 81·0]
	Complete GH response(mGH ≤ 2·5 µg/l)	42·6 (29) [30·1, 55·2]	87·5 (7) [47·4, 99·7]	36·7 (22) [23·5, 49·8]
	Partial response GH(mGH > 2·5 and ≤ 5 µg/l)	29·4 (20) [17·8, 41·1]	12·5 (1) [0·0, 52·7]	31·7 (19) [19·0, 44·4]
Any IGF-1 response		55·9 (38) [43·3, 68·5]	100·0 (8) [63·1, 100·0]	50·0 (30) [36·4, 63·6]
	Complete IGF-1 response(normal for age and sex)	38·2 (26) [25·9, 50·6]	75·0 (6) [34·9, 96·8]	33·3 (20) [20·5, 46·2]
	Partial IGF-1 response(≥ 50% reduction)	17·6 (12) [7·8, 27·5]	25·0 (2) [3·2, 65·1]	16·7 (10) [6·3, 27·0]
Week 48				
Any GH response		67·6 (46) [55·7, 79·6]	100·0 (8) [63·1, 100·0]	63·3 (38) [50·2, 76·5]
	Complete GH response(mGH ≤ 2·5 µg/l)	44·1 (30) [31·5, 56·7]	75·0 (6) [34·9, 96·8]	40·0 (24) [26·7, 53·3]
	Partial GH response(mGH > 2·5 and ≤ 5 µg/l)	23·5 (16) [12·6, 34·4]	25·0 (2) [3·2, 65·1]	23·3 (14) [11·7, 35·0]
Any IGF-1 response		48·5 (33) [35·8, 61·2]	75·0 (6) [34·9, 96·8]	45·0 (27) [31·5, 58·5]
	Complete IGF-1 response(normal for age and sex)	33·8 (23) [21·8, 45·9]	50·0 (4) [15·7, 84·3]	31·7 (19) [19·0, 44·4]
	Partial IGF-1 response(≥ 50% reduction)	14·7 (10) [5·5, 23·9]	25·0 (2) [3·2, 65·1]	13·3 (8) [3·8, 22·8]

* Using exact calculation method because of the small number of subjects.

**Table 4:** Treatment success rates

Week/Outcome	Total *N* = 68% (*n*) [95% CI]	Microadenomas *N* = 8% (*n*) [95% CI]*	Macroadenomas *N* = 60% (*n*) [95% CI]
Week 24			
Overall treatment success	50·0 (34)	100·0 (8)	43·3 (26)
	[37·3, 62·7]	[63·1, 100·0]	[29·9, 56·8]
Total success	26·5 (18)	75·0 (6)	20·0 (12)
	[15·2, 37·8]	[34·9, 96·8]	[9·0, 31·0]
Partial success	23·5 (16)	25·0 (2)	23·3 (14)
	[12·6, 34·4]	[3·2, 65·1]	[11·7, 35·0]
Week 48			
Overall treatment success	47·1 (32)	75·0 (6)	43·3 (26)
	[34·4, 59·7]	[34·9, 96·8]	[29·9, 56·8]
Total success	25·0 (17)	37·5 (3)	23·3 (14)
	[13·9, 36·1]	[8·5, 75·5]	[11·7, 35·0]
Partial success	22·1 (15)	37·5 (3)	20·0 (12)
	[11·4, 32·7]	[8·5, 75·5]	[9·0, 31·0]

Total success: mean of 2-h GH profile ≤ 2·5 µg/l and IGF-I levels within laboratory normal range.

Partial success: (1) mean of 2-h GH profile > 2·5 and ≤ 5·0 µg/l and either a decrease in IGF-I of at least 50% in comparison to baseline levels or IGF-I levels within laboratory normal range, or (2) mean of 2-h GH profile ≤ 2·5 µg/l and a decrease in IGF-I of at least 50% in comparison to baseline levels and IGF-I levels outside normal range.

* Using exact calculation method because of the small number of subjects.

### Tumour volume

**Fig. 3:**
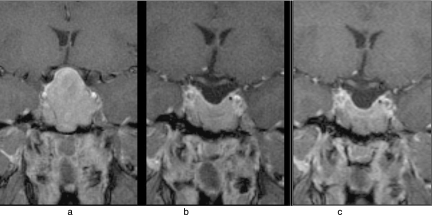
Coronal MR images in a patient with macroadenoma (a) at baseline (volume 22 702 mm^3^), (b) after 24 weeks (volume 15 285 mm^3^) and (c) after 48 weeks (volume 11 746 mm^3^) of treatment with octreotide LAR.

**Fig. 4:**
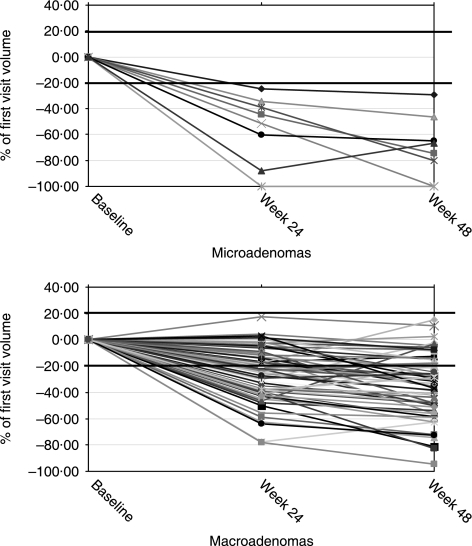
Percentages in tumour volume reduction in micro- and macroadenomas throughout the study.

Figure 3 illustrates tumour shrinkage as evidenced on serial MRI scans and Fig. 4 depicts individual percentages in tumour volume reduction throughout the study in micro- and macroadenomas. The magnitude of tumour volume reduction was greater in patients with microadenomas than in those with macroadenomas at both 24 and 48 weeks of treatment (week 24, *P* = 0·006; week 48, *P* = 0·002; Table 5). In the former group, tumour volume was reduced from a mean baseline level of 298 ± 145 mm^3^ to 139 ± 94 mm^3^ after 24 weeks of treatment and to 99 ± 70 mm^3^ after 48 weeks of treatment. In patients with macroadenoma, the values were 3885 ± 5077 mm^3^ at baseline, 2723 ± 3435 mm^3^ after 24 weeks and 2406 ± 3207 mm^3^ after 48 weeks. At week 24, 63% of patients (100% microadenoma and 58% macroadenoma, *P* = 0·02, Fisher's exact test) showed a substantial (≥ 20%) reduction in tumour volume whereas 75% (100% microadenoma and 72% macroadenoma, *P* = 0·19, Fisher's exact test) did so at week 48. Two patients with microadenoma had no visible tumour on MRI by week 48.

**Table 5:** Changes in tumour volume (expressed as negative percentages from baseline)

	Total *N* = 68	Microadenomas *N* = 8	Macroadenomas*N* = 60
Week 24			
*N*	68	8	60
Mean	−29	−55	−26
95% Confidence interval	[−35, −24]	[−70, −40]	[−32, −21]
Median	−28	−48	−24
SD	23·2	26·4	20·8
Minimum, Maximum	−18, −100	−24, −100	−18, −78
Week 48			
*N*	68	8	60
Mean	−39	−70	−35
95% Confidence interval	[−45, −32]	[−87, −53]	[−41, −29]
Median	−37	−70	−34
SD	26·4	24·5	23·9
Minimum, Maximum	−15, −100	−29, −100	−15, −94

A favourable biochemical response, be it the achievement of a GH ≤ 2·5 µg/l and/or the normalization of IGF-1, at 12 weeks was not predictive of a significant reduction in tumour volume by the end of the study. Approximately half of the initial biochemical responders and nonresponders showed greater than 20% tumour shrinkage by week 48. However, biochemical responders, in general, had significantly smaller tumours than nonresponders (1327 *vs.* 5362 mm^3^, *P* < 0·001).

### Signs and symptoms of acromegaly

As shown in Table 6, there was a marked and consistent reduction in the number and intensity of symptoms of acromegaly that was sustained throughout the treatment period.

**Table 6:** Incidence and severity of symptoms at baseline, week 24 and week 48

Symptom severity	Baseline *N* = 68 *n* (%)	Week 24 *N* = 68*n (%)	Week 48 *N* = 68 *n* (%)
Headache			
Absent	24 (35·3)	51 (76·1)	53 (77·9)
Mild	21 (30·9)	11 (16·4)	10 (14·7)
Moderate	15 (22·1)	5 (7·5)	4 (5·9)
Severe, not incapacitating	7 (10·3)	0	0
Severe, incapacitating	1 (1·5)	0	1 (1·5)
Fatigue			
Absent	25 (36·8)	46 (68·7)	52 (76·5)
Mild	23 (33·8)	16 (23·9)	12 (17·6)
Moderate	13 (19·1)	5 (7·5)	3 (4·4)
Severe, not incapacitating	7 (10·3)	0	0
Severe, incapacitating	0	0	1 (1·5)
Perspiration			
Absent	20 (29·4)	50 (74·6)	51 (75·0)
Mild	22 (32·4)	12 (17·9)	9 (13·2)
Moderate	18 (26·5)	3 (4·5)	7 (10·3)
Severe, not incapacitating	6 (8·8)	2 (3·0)	1 (1·5)
Severe, incapacitating	2 (2·9)	0	0
Osteoarthralgia			
Absent	17 (25·0)	35 (52·2)	38 (55·9)
Mild	18 (26·5)	22 (32·8)	19 (27·9)
Moderate	22 (32·4)	7 (10·4)	6 (8·8)
Severe, not incapacitating	9 (13·2)	3 (4·5)	5 (7·4)
Severe, incapacitating	2 (2·9)	0	0
Carpal tunnel syndrome			
Absent	47 (69·1)	62 (92·5)	66 (97·1)
Mild	13 (19·1)	3 (4·5)	0
Moderate	4 (5·9)	1 (1·5)	2 (2·9)
Severe, not incapacitating	2 (2·9)	1 (1·5)	0
Severe, incapacitating	2 (2·9)	0	0
Paresthesia			
Absent	33 (48·5)	61 (91·0)	60 (88·2)
Mild	19 (27·9)	5 (7·5)	7 (10·3)
Moderate	12 (17·6)	1 (1·5)	1 (1·5)
Severe, not incapacitating	3 (4·4)	0	0
Severe, incapacitating	1 (1·5)	0	0

* One patient was asymptomatic at week 24.

### Tolerability

The most frequently reported AEs were gastrointestinal complaints such as abdominal pain or discomfort, diarrhoea and nausea. Hepatobiliary disorders were also reported frequently and a total of 38 patients experienced newly occurring or worsening cholelithiasis. Baseline gallbladder ultrasound in 13 patients revealed at least one asymptomatic hepatobiliary abnormality (gallstones, sludge or bile duct dilation). The majority of cases of cholelithiasis were asymptomatic and required no intervention. One patient underwent a cholecystectomy because of acute cholecystitis but went on to complete the study. Two patients were prematurely withdrawn from the study because of clinically significant biliary disease, one of them with pancreatitis and cholestatic hepatitis.

## Discussion

This large multicentre, international study aimed to evaluate the safety and efficacy of octreotide LAR in the primary treatment of acromegaly. The participation of countries from Eastern and Western Europe, as well as Asia and Latin America, makes this a unique study in terms of its ethnic diversity. Ethnic factors can be important not only in the aetiology of acromegaly but also in determining the response to pharmacological therapy. The latter is illustrated by the differences in the prevalence of gsp alpha mutations among Caucasians,^19^,^20^ Asian^21^ and Mexican^22^ patients with acromegaly and the notion that tumours that harbour such molecular changes are less invasive and respond better to octreotide.^20^ After 48 weeks of treatment, 44% of our patients had achieved a safe GH level, 34% had normalized their IGF-1 levels and 25% achieved both criteria of biochemical control. This biochemical response rate is somewhat lower than the recently reported figures from two Italian groups,^23^,^24^ whereby a safe GH and a normal IGF-1 level are achieved by almost 60% of the patients. This discrepancy in outcome may be because these Italian studies included only Caucasian patients, whereas our study was carried out in subjects coming from several genetic backgrounds, including some with a low prevalence of gsp alpha mutations in somatotroph adenomas.^21^,^22^ Thus, our results derived from a large group of otherwise unselected patients, and by using stringent criteria for response, better represent the real success rate of somatostatin analogues in previously untreated acromegaly.

In general, the study drug was well tolerated. The most frequently reported AEs were gastrointestinal disorders including pain, discomfort, distension and diarrhoea. The inclusion of repeated echographic examinations of the gallbladder and biliary tree led to the detection of a relatively large number of cases of new or worsening cholelithiasis. Cholelithiasis is an AE that is known to be associated with octreotide therapy,^25^ and the majority of the cases were asymptomatic events that required no special interventions.

As in previously reported studies, we found that patients with microadenomas and also those with a lower degree of hypersomatotrophinaemia fared better with treatment in terms of both biochemical response rate and tumour volume reduction.^14^,^15^,^23^,^24^ In contrast to these other studies, however, we did not find an improvement in biochemical response with longer duration of treatment. In responsive patients the effects were detectable within 12 weeks of starting therapy and were maintained throughout the study. Amelioration of the biochemical abnormalities was accompanied by a consistent reduction in the incidence and severity of the symptoms of the disease including headache, fatigue, perspiration, paresthesia, osteoarthralgia and carpal tunnel syndrome, which also persisted throughout the whole study period.

In addition to suppressing the elevated GH and IGF-1 levels and controlling the signs and symptoms of the disease, octreotide LAR treatment led to a clear regression of tumour volumes (i.e. > 20% reduction from initial volume) in all the patients with microadenoma and in approximately 70% of those with macroadenoma. None of the patients showed an increase in tumour volume (i.e. outside the ± 20% measurement error). Even macroadenomas that did not exceed 20% shrinking still showed a steady reduction in size at 48 weeks. The microadenomas shrank rapidly, with the reduction in tumour volume being detectable after 24 weeks of therapy in all patients and the regression increased progressively over time to an average of 70% volume reduction after 48 weeks. Thus, continuing therapy with octreotide LAR may be appropriate in patients with macroadenoma even if the initial responses are small. Studies with much longer-term follow-up imaging (e.g. at least 2 years of octreotide LAR therapy) would be required to demonstrate the final time beyond which no further tumour shrinkage could be expected.

This is the largest study to date to use centralized volume measurement of electronic image data in acromegaly. The volume reductions found in the present study are consistent with the only previous study that systematically measured changes in tumour volume in 27 previously untreated GH-secreting tumours during octreotide therapy,^14^ despite differences in the measurement methods (summed area rather than diameter). While an estimate of volume can be obtained from lesion diameter measurements, these may not adequately account for irregularly shaped tumours that are frequent among macroadenomas, particularly large ones.^17^,^26^,^27^ Thus, very large macroadenomas (in the present study, 25% had a baseline volume of > 5000 mm^3^) are more difficult to measure than smaller lesions, even digitally. Our results strongly suggest that the larger the tumour the more slowly it shrinks. Of interest, tumour shrinkage was apparent even in a significant proportion of patients with a relatively poor biochemical response rate. This discrepancy between somatostatin analogue-induced tumour shrinkage and the ability of the compound to inhibit GH synthesis and secretion has been observed before in individual cases and appears to be due to the expression of a specific set of somatostatin receptor subtypes, particularly the sst3 subtype (as opposed to the usual expression in these tumours of subtypes 2 and 5).^28^,^29^

Thus, the results of this investigation confirm those published previously and demonstrate that octreotide LAR fulfils all the requirements for the primary treatment of acromegaly because it can reverse tumour growth, produce a sustained suppression of the elevated levels of GH and IGF-1 and control the symptoms of the disease.
